# Supplementary material for: Bi-directional regulation of cognitive control by distinct prefrontal cortical output neurons to thalamus and striatum
Source: Nat Commun. 2021 Mar 31;12:1994. doi: 10.1038/s41467-021-22260-7 (PMC8012364; doi:10.1038/s41467-021-22260-7)
Supplement: Supplementary file 1 — Supplementary Information [file 41467_2021_22260_MOESM1_ESM.pdf]

Supplementary figures

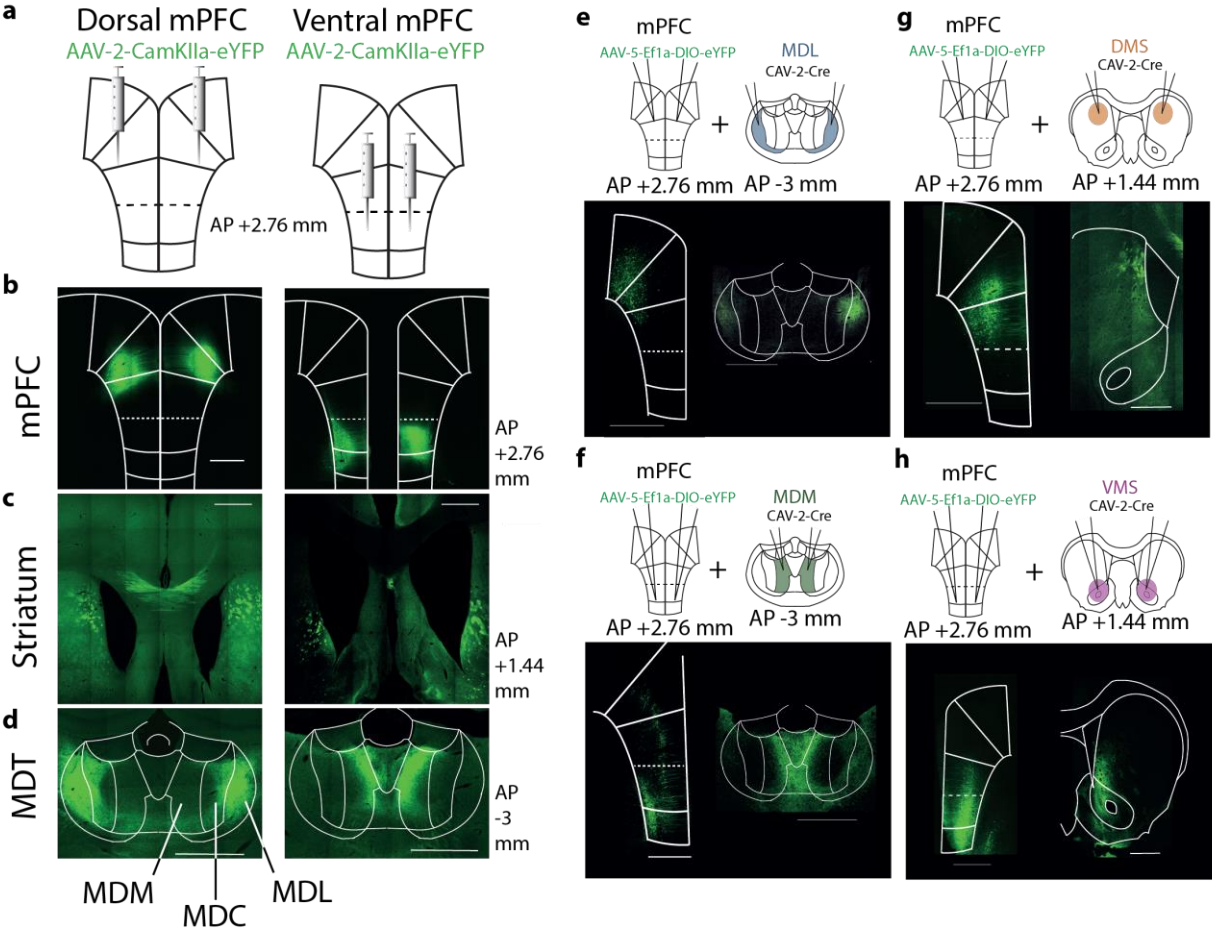

Supplementary Figure 1. Viral tracing in corticostriatal and corticothalamic projections.

a AAV2-CaMKIIa injection protocol for anterograde tracing experiments. **b** Prefrontal cortex eYFP expression following dorsal (left) and ventral (right) virus injections. **c** eYFP expression in corticostriatal axon terminals. Left: Injections and expression of eYFP in dorsal mPFC result in eYFP positive axon terminals in dorsal striatum. Right: Ventral mPFC eYFP expression leads to eYFP positive axon terminal fields in ventral striatum. **d** eYFP expression in corticothalamic axon terminals. Left: In the MDT, lateral portions (MDL) show positive axon terminals following injections and expression of eYFP in dorsal mPFC. Right: Medial portions of MDT show positive axon terminals after virus injection and eYFP expression in ventral mPFC. **e** Top: Cav2-Cre injection protocol for retrograde tracing MDL-projecting neurons. Bottom left: eYFP expression in dmPFC. Bottom right: eYFP expression in MDL. **f** Same as (**e**), but for MDM-projecting neurons. **g** Same as (**e**), but for DMS-projecting neurons. **h** Same as (**e**), but for VMS-projecting neurons. Scale bars in **b**-**h** 1 mm.

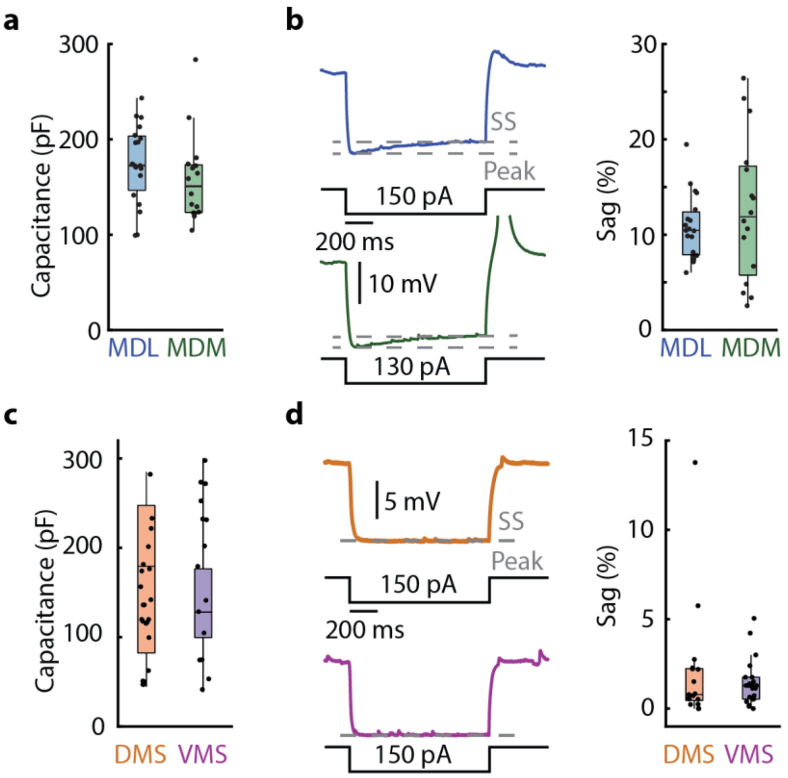

Supplementary Figure 2. Electrophysiological properties of dorsally and ventrally PFC-innervated MD and striatum neurons.
a Membrane capacitance in thalamic relay neurons. **b** Sag in thalamic relay neurons, calculated as percentage difference between Δ steady state (SS) and Δ peak from a hyperpolarizing current step resulting in a peak voltage change closest to -20mV. Left: example trace, right: summary plot. **c** Membrane capacitance in striatal medium spiny neurons. **d** same as (**b**), but for striatal medium spiny neurons. Boxplots: center line, median; box edges, 1st and 3rd quartile; whiskers, data range without outliers. Colors and group sizes: MDM (green, n = 18 cells, from 6 rats), MDL (blue, n = 19 cells, from 7 rats), VMS (purple, n = 21 cells, from 6 rats), DMS (orange, n = 23 cells, from 7 rats). Two-sided Mann-Whitney tests were used.

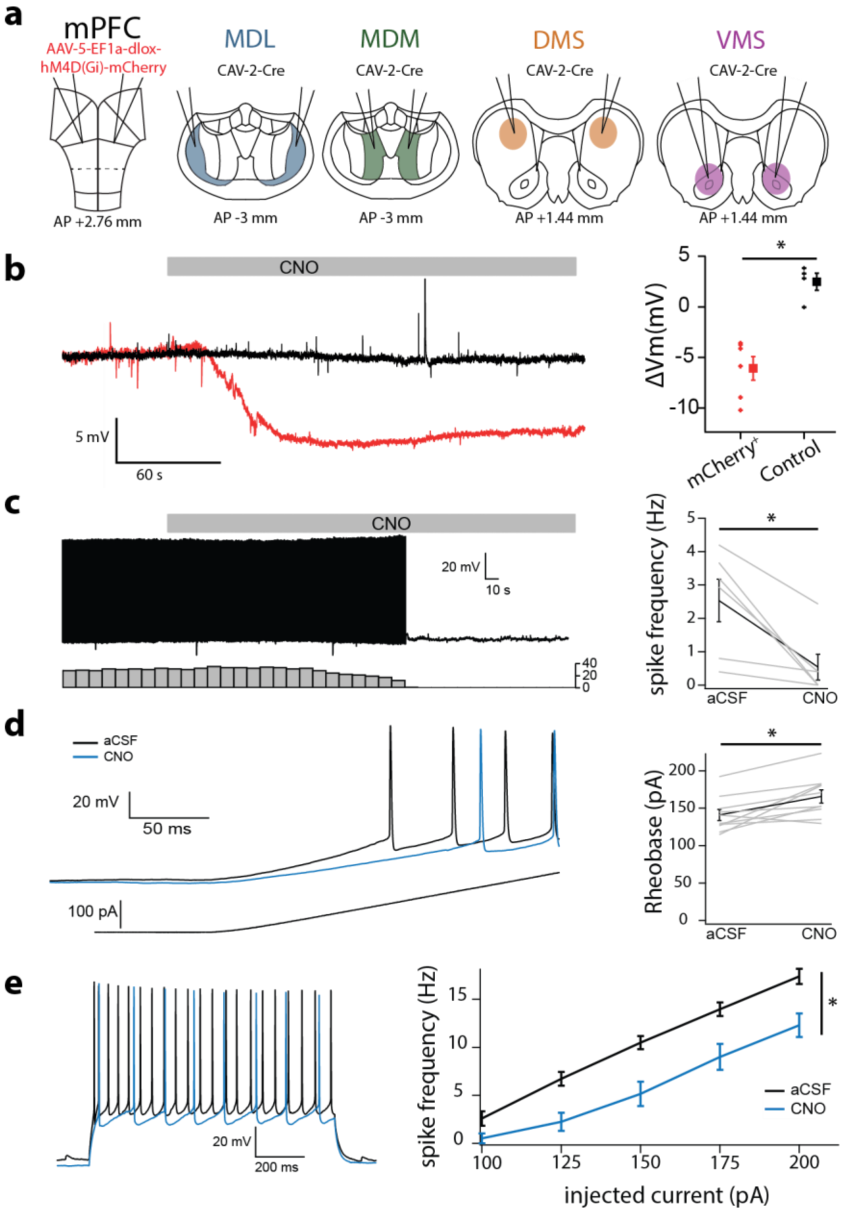

Supplementary Figure 3. DREADD-mediated hypofunction of specific PFC projection populations.

a Experimental design for expression of hM4D(Gi) receptors with mCherry-tag in prefrontal projection populations. **b** Left: raw traces from a mCherry-positive (red) and negative (black, control) neuron resting membrane potential before and during CNO bath application. Right: Quantification of CNO-induced resting membrane potential change. * p = 0.0095. mCherry^{+} n = 6 cells, control n = 4 cells. **c** Left top: Raw trace from mCherry+ neuron. Left bottom: Spike frequencies in 10s bins. Right: Quantification of spike frequency during aCSF or CNO application. * p = 0.015. n = 6 cells. **d** Left: raw trace of mCherry+ neuron during aCSF (black) and CNO (blue) bath application. 100 pA ramp current injection. Right: rheobase quantification. * p = 0.00079. n = 10 cells. **e** Spike frequency induced by different current steps. * $p < 0.01$. n = 10 cells. Error bars in **b-e** represent mean ± SEM. Two-sided Mann-Whitney test were used in **b** and **c**. Two-sided paired t-tests was used in **d**. Two-way ANOVA was used in **e**. Significance stars: * $p < 0.05$.

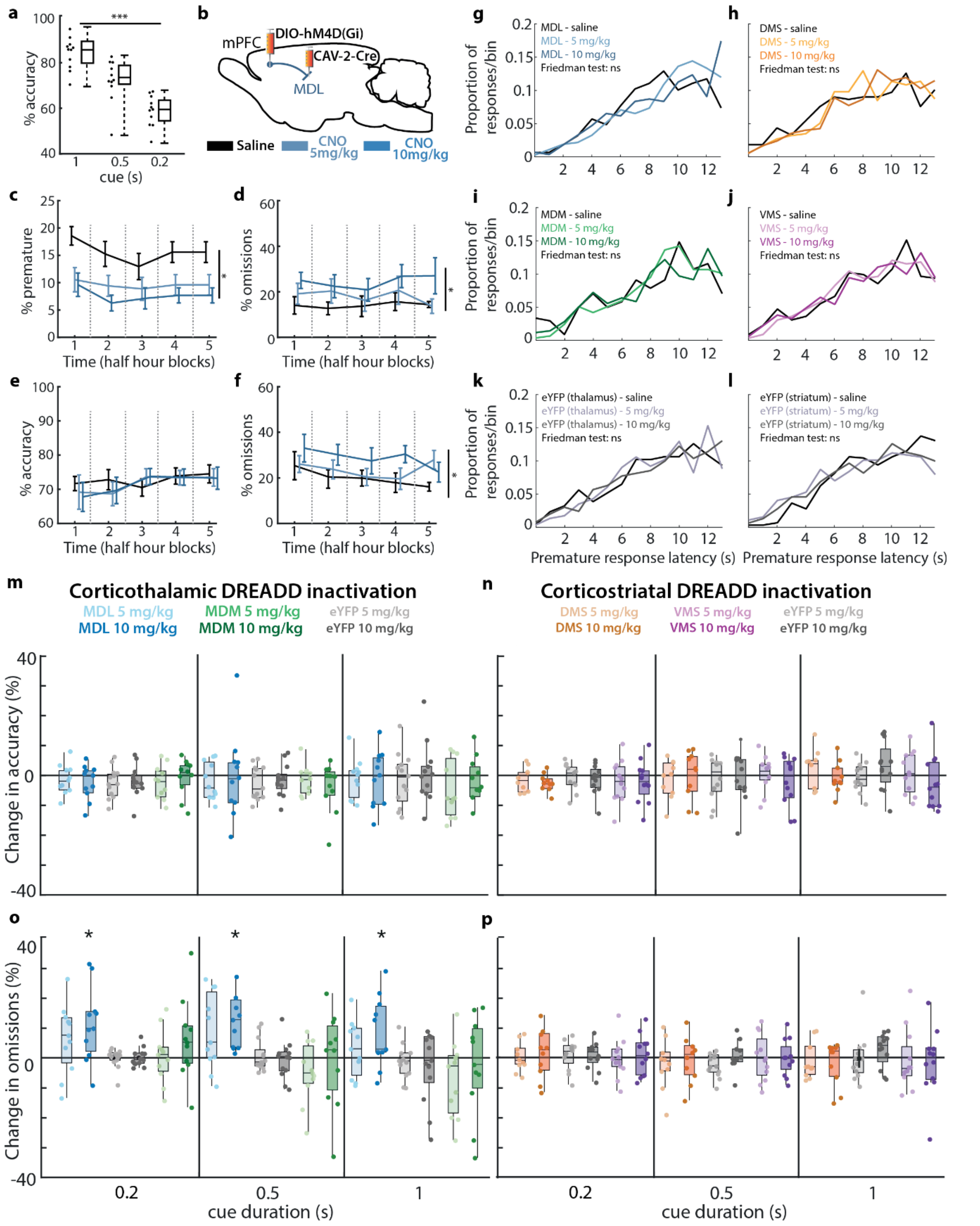

Supplementary Figure 4. Additional behavioral parameters after DREADD treatment during SP-5-CSRTT

a Accuracy in variable cue duration sessions. $F[2,20] = 27.76$, $p < 0.001$. **b** Virus injection protocol for expression of hM4D(Gi) receptor in MDL-projecting neurons. **c** Distribution of premature responses after saline and CNO injections in 2.5-hour variable delay sessions, divided into 30-minute blocks. $F[2,20] = 11.20$, $p < 0.001$. Only data for MDL-projecting neurons shown. **d** same as (**c**), for omissions in variable delay sessions. $F[2,20] = 8.00$, $p = 0.002$. **e** same as (**c**), for accuracy in variable cue duration sessions. **f** same as (**d**), for omissions in variable cue duration sessions. **g-l** Proportion of premature responses during 1s

bins in 12.5s-delay trials. **m** Difference in accuracy between CNO sessions and saline during variable cue duration sessions in mPFC-MD projections. **n** Same as (**m**), but for mPFC-striatum projections. **o-p** Similar as (**m**-**n**) but for change in omissions. MDL: $F[4,64] = 3.71$, $p = 0.008$. Dots represent individual rats in panels **a, m-p**. Boxplots in **a**, **m**-**p**: center line, median; box edges, 1st and 3th quartile; whiskers, data range without outliers. Error bars in **c**-**f** represent mean±SEM. One-way ANOVA was used in **a**. Repeated-measures ANOVA was used in **c**-**f**. Friedman tests were done in **g**-**l**. Three-way mixed repeated-measures ANOVA with post-hoc paired two-sided t-tests compared to saline baseline were used in **m**-**p**. Significance: * $p < 0.05$, *** $p < 0.001$. Group sizes and colors: MDL ($n = 11$), MDM ($n = 11$), eYFP Thalamus ($n = 13$, panels **m**, **o**) DMS ($n = 10$, orange), VMS ($n = 12$, purple), eYFP Striatum ($n = 12$, grey, panels **n**, **p**).

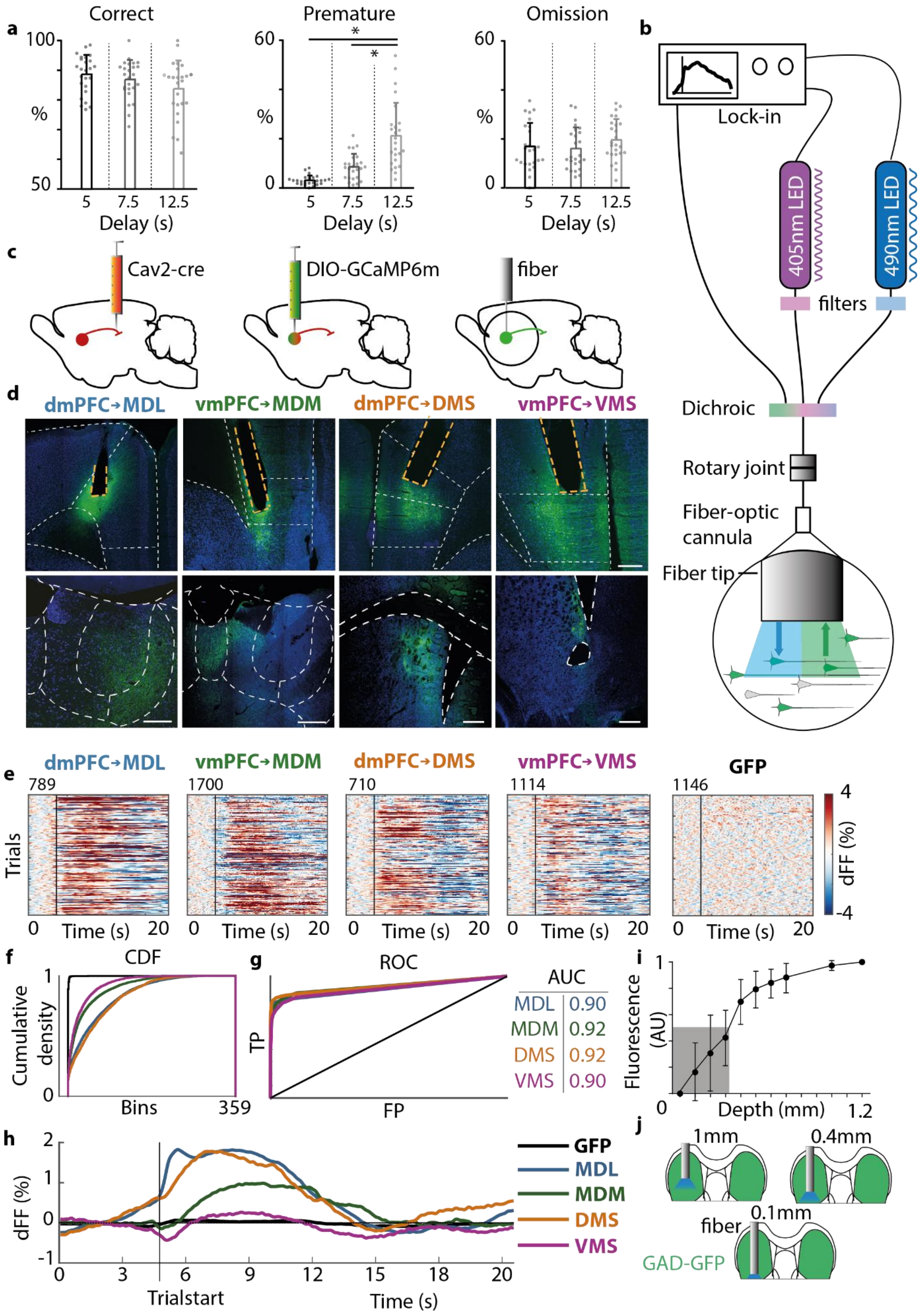

Supplementary Figure 5. Behavioral parameters, equipment, viral expression and signal dynamics during fiber photometry experiments.

a 5-CSRTT performance parameters during fiber photometry recording sessions. $F[2,72] = 29.24$, $p < 0.0001$, $Delay_{short}$ vs. $Delay_{long}$ $p < 0.0001$, $Delay_{mid}$ vs. $Delay_{long}$ $p < 0.0001$. Dots represent individual rats. Bar and data point colors represent delay duration. $n = 25$ rats. **b** Schematic representation of fiber photometry setup. **c** Experimental procedure for GCaMP6m expression in prefrontal projection populations. **d** Top: Representative examples of GCaMP6m expression in somata of prefrontal projection neurons. White dashed line: Brain atlas overlay. Yellow dashed lines: fiber tract. Scale bar 500μm. Bottom: Examples of GCaMP6m expression in axon terminals in target areas. White dashed lines: Brain atlas overlay. Scale bars: 500μm. **e** Example heatmaps for animals with either GFP (left), or GCaMP6m expressed in projection populations. Only sessions with variable delay, but all trial outcomes and delays have been pooled together. Data are z-scored to baseline (t-5 to t-1 from trialstart). **f** Cumulative density function (CDF) for bins that are >2std above or below baseline. X axis is number of bins, Y axis is cumulative proportion of trials. **g** Receiver operator characteristic for trials made by GFP-expressing rats vs each projection population. TP: True positives, FP: False positives, AUC: area under curve. **h** Population averages for GFP rats and projection populations. **i** Experimental procedure for determination of excitation light tissue coverage. $n = 4$ brains. **j** Proportion of fluorescence compared to maximum fluorescence measured in recording session. Grey area corresponds to 50% of maximum signal. Error bars in **a** represent mean ± SEM. Error bars in **i** represent mean ± SD. One-way ANOVA with Tukey post-hoc test was used in **a**. Significance: * $p < 0.01$. Color scale in **e** is the same for each heat plot.

Supplementary Tables

Table S1. Behavioral performance during variable delay sessions in rats expressing hM4D(Gi) in dmPFC→MDL projecting neurons.

Summary of behavioral parameters from variable delay shown per delay duration.
CNO_5: CNO 5 mg/kg. CNO_{10}: CNO 10 mg/kg injection.
Data are expressed as mean ± SD.

**dmPFC→MDL**	Delay (s)	Saline	CNO_5	CNO_{10}
Accuracy (%)	5<br>7.5<br>12.5	86.6 ± 8.59<br>85.4 ± 7.04<br>80.46 ± 6.39	88.51 ± 6.22<br>86.80 ± 6.18<br>80.67 ± 7.57	90.88 ± 7.44<br>87.07 ± 7.60<br>81.61 ± 6.22
Premature responses (%)	5<br>7.5<br>12.5	2.38 ± 1.48<br>14.70 ± 7.21<br>44.78 ± 17.15	1.37 ± 1.35<br>9.88 ± 6.16<br>27.99 ± 16.67	1.81 ± 1.89<br>6.35 ± 4.76<br>22.63 ± 15.31
Omissions (%)	5<br>7.5<br>12.5	18.92 ± 14.55<br>11.24 ± 8.82<br>9.19 ± 5.76	21.48 ± 17.83<br>17.90 ± 11.70<br>11.28 ± 7.43	30.12 ± 19.30<br>22.91 ± 14.59<br>17.90 ± 11.22
Correct response latency (s)	5<br>7.5<br>12.5	1.06 ± 0.20<br>0.98 ± 0.19<br>0.96 ± 0.19	1.04 ± 0.17<br>1.02 ± 0.19<br>0.97 ± 0.16	1.12 ± 0.22<br>1.06 ± 0.20<br>1.02 ± 0.19
Premature response latency (s)	5<br>7.5<br>12.5	3.81 ± 0.41<br>5.67 ± 0.48<br>8.72 ± 0.47	4.20 ± 0.35<br>5.76 ± 0.60<br>9.22 ± 0.47	3.70 ± 0.69<br>5.50 ± 0.65<br>9.10 ± 1.09

Table S2. Behavioral performance during variable delay sessions in rats expressing hM4D(Gi) in vmPFC→MDM projecting neurons.

Summary of behavioral parameters from variable delay shown per delay duration.
CNO5: CNO 5 mg/kg. CNO10: CNO 10 mg/kg injection.
Data are expressed as mean ± SD.

**vmPFC→MDM**	Delay (s)	Saline	CNO_5	CNO_{10}
Accuracy (%)	5<br>7.5<br>12.5	88.54 ± 9.13<br>85.57 ± 11.11<br>81.36 ± 12.58	85.27 ± 12.81<br>83.21 ± 9.29<br>78.52 ± 14.40	84.97 ± 11.59<br>84.03 ± 11.33<br>80.34 ± 13.35
Premature responses (%)	5<br>7.5<br>12.5	3.83 ± 3.85<br>8.37 ± 5.95<br>21.09 ± 7.93	4.01 ± 2.56<br>10.90 ± 6.03<br>28.02 ± 11.86	2.85 ± 3.48<br>8.84 ± 6.10<br>30.26 ± 10.28
Omissions (%)	5<br>7.5<br>12.5	27.66 ± 21.54<br>20.13 ± 15.10<br>15.60 ± 10.80	29.38 ± 19.24<br>21.97 ± 15.15<br>16.34 ± 13.44	29.63 ± 22.70<br>23.88 ± 18.32<br>14.41 ± 10.26
Correct response latency (s)	5<br>7.5<br>12.5	1.24 ± 0.38<br>1.15 ± 0.34<br>1.02 ± 0.29	1.22 ± 0.39<br>1.09 ± 0.28<br>1.00 ± 0.17	1.23 ± 0.38<br>1.14 ± 0.30<br>1.04 ± 0.24
Premature response latency (s)	5<br>7.5<br>12.5	3.83 ± 0.50<br>5.72 ± 0.98<br>8.71 ± 1.26	3.57 ± 0.76<br>5.43 ± 0.62<br>8.61 ± 1.40	3.76 ± 0.82<br>5.41 ± 0.98<br>8.90 ± 1.02

Table S3. Behavioral performance during variable delay sessions in rats expressing eYFP in thalamus-projecting neurons.

Summary of behavioral parameters from variable delay shown per delay duration.
CNO5: CNO 5 mg/kg. CNO10: CNO 10 mg/kg injection.
Data are expressed as mean ± SD.

**eYFP (Thalamus)**	Delay (s)	Saline	CNO_5	CNO_{10}
Accuracy (%)	5<br>7.5<br>12.5	91.45 ± 7.27<br>87.29 ± 9.42<br>85.09 ± 7.77	91.45 ± 7.27<br>87.29 ± 9.42<br>85.09 ± 7.77	91.45 ± 7.27<br>87.29 ± 9.42<br>85.09 ± 7.77
Premature responses (%)	5<br>7.5<br>12.5	4.17 ± 2.94<br>12.20 ± 7.26<br>31.76 ± 18.17	4.17 ± 2.94<br>12.20 ± 7.26<br>31.76 ± 18.17	4.17 ± 2.94<br>12.20 ± 7.26<br>31.76 ± 18.17
Omissions (%)	5<br>7.5<br>12.5	8.01 ± 5.41<br>7.20 ± 5.79<br>4.88 ± 3.91	8.01 ± 5.41<br>7.20 ± 5.79<br>4.88 ± 3.91	8.01 ± 5.41<br>7.20 ± 5.79<br>4.88 ± 3.91
Correct response latency (s)	5<br>7.5<br>12.5	0.80 ± 0.20<br>0.78 ± 0.20<br>0.81 ± 0.20	0.80 ± 0.20<br>0.78 ± 0.20<br>0.81 ± 0.20	0.80 ± 0.20<br>0.78 ± 0.20<br>0.81 ± 0.20
Premature response latency (s)	5<br>7.5<br>12.5	4.06 ± 0.43<br>5.27 ± 0.59<br>8.67 ± 0.70	4.06 ± 0.43<br>5.27 ± 0.59<br>8.67 ± 0.70	4.06 ± 0.43<br>5.27 ± 0.59<br>8.67 ± 0.70

Table S4. Behavioral performance during variable delay sessions in rats expressing hM4D(Gi) in dmPFC→DMS projecting neurons.

Summary of behavioral parameters from variable cue duration sessions shown per cue duration.
CNO5: CNO 5 mg/kg. CNO10: CNO 10 mg/kg injection.
Data are expressed as mean ± SD.

**dmPFC→DMS**	Delay (s)	Saline	CNO_5	CNO_{10}
Accuracy (%)	5<br>7.5<br>12.5	88.09 ±2.56<br>88.56 ±3.62<br>84.85 ±6.80	89.38 ±4.54<br>86.46 ±4.00<br>82.84 ±7.29	87.99 ±5.44<br>87.33 ±5.39<br>82.03 ±6.94
Premature responses (%)	5<br>7.5<br>12.5	2.88 ±1.91<br>6.99 ±4.69<br>19.80 ±12.07	3.47 ±2.30<br>9.40 ±4.72<br>26.38 ±13.84	3.05 ±1.92<br>9.70 ±6.06<br>29.74 ±12.64
Omissions (%)	5<br>7.5<br>12.5	12.35 ±6.23<br>8.54 ±3.77<br>9.13 ±6.12	10.06 ±5.97<br>6.38 ±4.37<br>8.80 ±4.56	7.97 ±3.27<br>7.22 ±5.10<br>9.71 ±5.45
Correct response latency (s)	5<br>7.5<br>12.5	1.26 ±0.16<br>1.19 ±0.17<br>1.15 ±0.14	1.21 ±0.22<br>1.13 ±0.16<br>1.13 ±0.16	1.16 ±0.12<br>1.14 ±0.12<br>1.11 ±0.12
Premature response latency (s)	5<br>7.5<br>12.5	3.64 ±0.55<br>5.51 ±0.75<br>8.18 ±0.68	3.41 ±0.51<br>5.32 ±0.53<br>8.35 ±0.71	3.51 ±0.61<br>5.52 ±0.45<br>8.62 ±0.47

Table S5. Behavioral performance during variable delay sessions in rats expressing hM4D(Gi) in vmPFC→VMS projecting neurons.

Summary of behavioral parameters from variable cue duration sessions shown per cue duration.
CNO5: CNO 5 mg/kg. CNO10: CNO 10 mg/kg injection.
Data are expressed as mean ± SD.

**vmPFC→VMS**	Delay (s)	Saline	CNO_5	CNO_{10}
Accuracy (%)	5<br>7.5<br>12.5	89.82 ±6.03<br>89.04 ±8.41<br>81.59 ±10.23	89.13 ±5.56<br>88.16 ±7.39<br>83.86 ±9.13	91.65 ±4.98<br>88.43 ±7.06<br>82.67 ±9.37
Premature responses (%)	5<br>7.5<br>12.5	3.63 ±2.55<br>8.30 ±5.76<br>25.22 ±15.88	3.92 ±2.20<br>11.29 ±5.53<br>28.15 ±14.38	3.46 ±2.98<br>9.79 ±5.17<br>26.82 ±14.15
Omissions (%)	5<br>7.5<br>12.5	7.25 ±3.96<br>6.95 ±3.34<br>8.24 ±5.65	9.25 ±4.72<br>5.97 ±2.33<br>7.11 ±4.59	8.25 ±5.43<br>6.51 ±5.03<br>6.36 ±2.87
Correct response latency (s)	5<br>7.5<br>12.5	1.12 ±0.17<br>1.09 ±0.19<br>1.08 ±0.16	1.12 ±0.18<br>1.04 ±0.17<br>1.05 ±0.18	1.10 ±0.19<br>1.03 ±0.15<br>1.01 ±0.16
Premature response latency (s)	5<br>7.5<br>12.5	3.42 ±0.58<br>5.36 ±0.46<br>8.36 ±0.64	3.75 ±0.56<br>5.41 ±0.58<br>8.32 ±0.83	3.46 ±0.46<br>5.42 ±0.50<br>8.57 ±0.85

Table S6. Behavioral performance during variable delay sessions in rats expressing eYFP in striatum-projecting neurons.

Summary of behavioral parameters from variable cue duration sessions shown per cue duration.
CNO5: CNO 5 mg/kg. CNO10: CNO 10 mg/kg injection.
Data are expressed as mean ± SD.

**eYFP (Striatum)**	Delay (s)	Saline	CNO_5	CNO_{10}
Accuracy (%)	5<br>7.5<br>12.5	89.94 ±6.17<br>88.62 ±8.05<br>80.90 ±9.80	89.13 ±5.56<br>88.16 ±7.39<br>83.86 ±9.13	91.53 ±4.85<br>88.85 ±7.49<br>83.36 ±9.69
Premature responses (%)	5<br>7.5<br>12.5	4.00 ±2.92<br>9.21 ±6.80<br>27.21 ±16.29	3.92 ±2.20<br>11.29 ±5.53<br>28.15 ±14.38	3.09 ±2.54<br>8.88 ±3.85<br>24.83 ±13.62
Omissions (%)	5<br>7.5<br>12.5	7.18 ±3.96<br>6.46 ±2.89<br>8.05 ±5.72	9.25 ±4.72<br>5.97 ±2.32<br>7.11 ±4.59	8.31 ±5.41<br>6.99 ±5.30<br>6.54 ±2.87
Correct response latency (s)	5<br>7.5<br>12.5	1.10 ±0.15<br>1.07 ±0.18<br>1.07 ±0.15	1.12 ±0.18<br>1.04 ±0.17<br>1.05 ±0.18	1.11 ±0.20<br>1.05 ±0.17<br>1.02 ±0.17
Premature response latency (s)	5<br>7.5<br>12.5	3.50 ±0.59<br>5.39 ±0.47<br>8.33 ±0.64	3.75 ±0.56<br>5.41 ±0.58<br>8.32 ±0.83	3.37 ±0.42<br>5.39 ±0.50<br>8.61 ±0.84

Table S7 Effects of chemogenetic corticothalamic inactivations on general task parameters.

Summary of general behavioral parameters from variable delay and cue duration sessions. CNO_5: CNO 5 mg/kg. CNO_{10}: CNO 10 mg/kg injection.
Data are expressed as mean ± SD.

		dmPFC→MDL			vmPFC→MDM			eYFP		
Parameter	Sess. type	Saline	CNO_5	CNO_{10}	Saline	CNO_5	CNO_{10}	Saline	CNO_5	CNO_{10}
Number of started trials	Var delay	349.91 ±84.28	328.18 ±78.36	304.36 ±82.85	298.27 ±72.78	306.18 ±102.95	310.36 ±94.32	363.31 ±125.62	398.77 ±93.44	396.08 ±87.77
Magazine latency (s)	Var delay	2.37 ±0.83	2.45 ±0.96	2.47 ±0.97	2.38 ±0.39	2.20 ±0.35	2.30 ±0.39	2.23 ±0.47	2.18 ±0.47	2.20 ±0.51
Perseve-rative responses on target (%)	Var delay	5.09 ±3.01	5.27 ±2.80	5.35 ±3.38	5.69 ±6.44	4.70 ±5.09	5.67 ±5.36	4.56 ±3.05	4.34 ±2.86	5.05 ±2.69
Number of started trials	Var cue	382.36 ±103.93	371.82 ±108.20	346.18 ±104.22	351.18 ±108.78	381.00 ±105.98	334.73 ±103.48	462.54 ±133.05	463.38 ±134.72	449.46 ±133.68
Magazine latency (s)	Var cue	2.53 ±1.11	2.48 ±1.24	2.42 ±0.96	2.27 ±0.34	2.17 ±0.31	2.17 ±0.41	2.10 ±0.35	2.18 ±0.50	2.22 ±0.48
Perseve-rative responses on target (%)	Var cue	4.21 ±2.16	3.66 ±2.74	4.37 ±3.55	4.26 ±5.55	4.24 ±5.41	3.51 ±3.85	4.21 ±2.82	4.01 ±2.53	3.49 ±2.20

Table S8. Behavioral performance during variable cue duration sessions in rats expressing hM4D(Gi) in dmPFC→MDL projecting neurons.

Summary of behavioral parameters from variable cue duration sessions shown per cue duration.
CNO5: CNO 5 mg/kg. CNO10: CNO 10 mg/kg injection.
Data are expressed as mean ± SD.

**dmPFC→MDL**	Cue (s)	Saline	CNO_5	CNO_{10}
Accuracy (%)	1<br>0.5<br>0.2	84.01 ± 7.00<br>72.00 ± 9.52<br>58.64 ± 6.79	82.70 ± 7.51<br>70.20 ± 9.17<br>57.65 ± 5.64	81.78 ± 8.98<br>71.95 ± 9.83<br>57.60 ± 12.32
Premature responses (%)	1<br>0.5<br>0.2	3.77 ± 3.39<br>4.70 ± 4.19<br>4.10 ± 3.68	3.38 ± 2.73<br>4.13 ± 4.13<br>4.81 ± 2.91	3.36 ± 1.80<br>3.39 ± 2.35<br>3.56 ± 1.73
Omissions (%)	1<br>0.5<br>0.2	19.28 ± 12.86<br>25.93 ± 13.33<br>40.39 ± 16.37	25.50 ± 14.40<br>34.60 ± 16.90<br>43.32 ± 15.93	30.32 ± 15.31<br>39.99 ± 13.38<br>48.58 ±16.71
Correct response latency (s)	1<br>0.5<br>0.2	0.99 ± 0.22<br>0.90 ± 0.21<br>0.84 ± 0.17	1.04 ± 0.21<br>0.92 ± 0.21<br>0.91 ± 0.18	1.08 ± 0.27<br>0.95 ± 0.21<br>0.87 ± 0.18
Accuracy (%)	1<br>0.5<br>0.2	84.01 ± 7.00<br>72.00 ± 9.52<br>58.64 ± 6.79	82.70 ± 7.51<br>70.20 ± 9.17<br>57.65 ± 5.64	81.78 ± 8.98<br>71.95 ± 9.83<br>57.60 ± 12.32

Table S9. Behavioral performance during variable cue duration sessions in rats expressing hM4D(Gi) in vmPFC→MDM projecting neurons.

Summary of behavioral parameters from variable cue duration sessions shown per cue duration.
CNO5: CNO 5 mg/kg. CNO10: CNO 10 mg/kg injection.
Data are expressed as mean ± SD.

vmPFC→MDM	Cue (s)	Saline	CNO_5	CNO_{10}
Accuracy (%)	1<br>0.5<br>0.2	82.24 ± 11.74<br>72.17 ± 11.00<br>61.70 ± 14.96	79.89 ± 8.35<br>70.88 ± 9.88<br>56.98 ± 8.45	81.75 ± 12.17<br>68.29 ± 12.52<br>57.03 ± 19.01
Premature responses (%)	1<br>0.5<br>0.2	5.53 ± 5.49<br>5.24 ± 4.86<br>5.17 ± 4.48	6.53 ± 4.27<br>6.53 ± 3.98<br>5.36 ± 3.05	7.42 ± 5.62<br>5.34 ± 3.65<br>5.77 ± 3.66
Omissions (%)	1<br>0.5<br>0.2	22.79 ± 18.69<br>34.17 ± 22.06<br>46.55 ± 25.77	23.21 ± 19.29<br>29.09 ± 21.36<br>39.28 ± 21.42	28.71 ± 24.95<br>33.83 ± 25.03<br>43.75 ± 27.09
Correct response latency (s)	1<br>0.5<br>0.2	1.14 ± 0.37<br>0.98 ± 0.27<br>0.86 ± 0.24	1.05 ± 0.29<br>0.93 ± 0.25<br>0.86 ± 0.23	1.09 ± 0.34<br>0.95 ± 0.34<br>0.86 ± 0.25
Premature response latency (s)	1<br>0.5<br>0.2	82.24 ± 11.74<br>72.17 ± 11.00<br>61.70 ± 14.96	79.89 ± 8.35<br>70.88 ± 9.88<br>56.98 ± 8.45	81.75 ± 12.17<br>68.29 ± 12.52<br>57.03 ± 19.01

Table S10. Behavioral performance during variable cue duration sessions in rats expressing eYFP in thalamus-projecting neurons.

Summary of behavioral parameters from variable cue duration sessions shown per cue duration.
CNO5: CNO 5 mg/kg. CNO10: CNO 10 mg/kg injection.
Data are expressed as mean ± SD.

**eYFP (Thalamus)**	Cue (s)	Saline	CNO_5	CNO_{10}
Accuracy (%)	1<br>0.5<br>0.2	90.40 ±6.35<br>78.29 ±9.76<br>61.41 ±6.6	88.02 ±6.95<br>75.76 ±10.56<br>60.07 ±11.20	88.35 ±6.32<br>76.72 ±10.92<br>62.21 ±10.56
Premature responses (%)	1<br>0.5<br>0.2	7.34 ± 5.59<br>5.91 ± 3.54<br>6.65 ± 4.26	7.32 ± 5.66<br>6.91 ± 4.89<br>7.39 ± 4.61	8.64 ± 6.16<br>7.45 ± 5.60<br>7.23 ± 5.99
Omissions (%)	1<br>0.5<br>0.2	6.27 ± 6.30<br>11.51 ± 6.60<br>22.52 ± 12.40	6.07 ± 4.47<br>12.08 ± 9.01<br>21.01 ± 9.44	6.26 ± 6.49<br>10.75 ± 6.45<br>19.72 ± 10.89
Correct response latency (s)	1<br>0.5<br>0.2	0.76 ± 0.17<br>0.65 ± 0.13<br>0.60 ± 0.17	0.76 ± 0.18<br>0.67 ± 0.15<br>0.59 ± 0.14	0.74 ± 0.20<br>0.67 ± 0.15<br>0.60 ± 0.15
Premature response latency (s)	1<br>0.5<br>0.2	90.40 ±6.35<br>78.29 ±9.76<br>61.41 ±6.6	88.02 ±6.95<br>75.76 ±10.56<br>60.07 ±11.20	88.35 ±6.32<br>76.72 ±10.92<br>62.21 ±10.56

Table S11. Behavioral performance during variable cue duration sessions in rats expressing hM4D(Gi) in dmPFC→DMS projecting neurons.

Summary of behavioral parameters from variable cue duration sessions shown per cue duration. CNO5: CNO 5 mg/kg. CNO10: CNO 10 mg/kg injection.
Data are expressed as mean ± SD.

**dmPFC→DMS**	Cue (s)	Saline	CNO_5	CNO_{10}
Accuracy (%)	1<br>0.5<br>0.2	87.38 ±3.82<br>70.94 ±7.11<br>60.73 ±10.44	85.94 ±5.06<br>69.72 ±6.91<br>63.13 ±12.06	84.97 ±4.73<br>70.89 ±7.00<br>60.02 ±9.29
Premature responses (%)	1<br>0.5<br>0.2	3.12 ±2.06<br>3.49 ±1.88<br>3.50 ±2.69	4.44 ±1.42<br>3.33 ±3.29<br>3.79 ±2.56	3.81 ±2.68<br>2.92 ±2.37<br>3.99 ±2.80
Omissions (%)	1<br>0.5<br>0.2	15.20 ±6.41<br>22.73 ±9.42<br>33.78 ±11.88	15.07 ±5.24<br>20.42 ±8.72<br>32.41 ±11.69	16.13 ±5.91<br>21.81 ±8.90<br>31.65 ±7.55
Correct response latency (s)	1<br>0.5<br>0.2	1.32 ±0.15<br>1.09 ±0.13<br>0.99 ±0.16	1.29 ±0.16<br>1.06 ±0.14<br>0.93 ±0.15	1.27 ±0.19<br>1.08 ±0.17<br>0.94 ±0.13
Premature response latency (s)	1<br>0.5<br>0.2	87.38 ±3.82<br>70.94 ±7.11<br>60.73 ±10.44	85.94 ±5.06<br>69.72 ±6.91<br>63.13 ±12.06	84.97 ±4.73<br>70.89 ±7.00<br>60.02 ±9.29

Table S12. Behavioral performance during variable cue duration sessions in rats expressing hM4D(Gi) in vmPFC→VMS projecting neurons.

Summary of behavioral parameters from variable cue duration sessions shown per cue duration.
CNO5: CNO 5 mg/kg. CNO10: CNO 10 mg/kg injection.
Data are expressed as mean ± SD.

**vmPFC→VMS**	Delay (s)	Saline	CNO_5	CNO_{10}
Accuracy (%)	1<br>0.5<br>0.2	87.35 ±5.66<br>70.83 ±6.68<br>60.25 ±9.74	85.30 ±7.40<br>71.48 ±7.15<br>61.24 ±12.41	85.30 ±6.79<br>68.80 ±10.92<br>57.85 ±11.92
Premature responses (%)	1<br>0.5<br>0.2	3.81 ±4.49<br>3.19 ±4.05<br>2.87 ±2.04	4.30 ±3.71<br>4.04 ±3.76<br>4.20 ±3.73	6.07 ±6.83<br>6.53 ±8.13<br>6.25 ±7.84
Omissions (%)	1<br>0.5<br>0.2	14.41 ±12.18<br>21.33 ±11.27<br>33.00 ±12.41	13.91 ±13.38<br>21.11 ±14.85<br>31.93 ±18.42	14.86 ±10.93<br>21.02 ±11.98<br>31.33 ±15.04
Correct response latency (s)	1<br>0.5<br>0.2	1.25 ±0.32<br>1.05 ±0.26<br>0.98 ±0.22	1.23 ±0.33<br>1.07 ±0.30<br>0.95 ±0.23	1.21 ±0.32<br>1.02 ±0.23<br>0.93 ±0.26
Premature response latency (s)	1<br>0.5<br>0.2	87.35 ±5.66<br>70.83 ±6.68<br>60.25 ±9.74	85.30 ±7.40<br>71.48 ±7.15<br>61.24 ±12.41	85.30 ±6.79<br>68.80 ±10.92<br>57.85 ±11.92

Table S13. Behavioral performance during variable cue duration sessions in rats expressing eYFP in striatum-projecting neurons.

Summary of behavioral parameters from variable cue duration sessions shown per cue duration.
CNO5: CNO 5 mg/kg. CNO10: CNO 10 mg/kg injection.
Data are expressed as mean ± SD.

**eYFP (Striatum)**	Delay (s)	Saline	CNO_5	CNO_{10}
Accuracy (%)	1<br>0.5<br>0.2	90.02 ±5.00<br>78.13 ±4.93<br>65.13 ±4.87	89.52 ±6.11<br>77.12 ±6.05<br>64.49 ±6.43	88.78 ±4.66<br>77.60 ±6.74<br>68.24 ±8.03
Premature responses (%)	1<br>0.5<br>0.2	4.24 ±2.44<br>4.03 ±3.02<br>4.51 ±3.17	4.25 ±3.85<br>4.42 ±3.56<br>4.04 ±3.59	4.00 ±2.70<br>4.66 ±3.21<br>3.03 ±2.00
Omissions (%)	1<br>0.5<br>0.2	6.80 ±4.56<br>11.44 ±6.77<br>17.73 ±6.37	7.54 ±5.28<br>8.55 ±5.31<br>17.42 ±9.51	7.32 ±3.55<br>11.83 ±6.07<br>20.10 ±7.15
Correct response latency (s)	1<br>0.5<br>0.2	1.09 ±0.15<br>0.94 ±0.14<br>0.86 ±0.15	1.10 ±0.14<br>0.94 ±0.09<br>0.85 ±0.12	1.10 ±0.12<br>0.96 ±0.10<br>0.89 ±0.08
Premature response latency (s)	1<br>0.5<br>0.2	90.02 ±5.00<br>78.13 ±4.93<br>65.13 ±4.87	89.52 ±6.11<br>77.12 ±6.05<br>64.49 ±6.43	88.78 ±4.66<br>77.60 ±6.74<br>68.24 ±8.03

Table S14 Effects of chemogenetic cortico-striatal inactivations on general task parameters. Summary of general behavioral parameters from variable delay and cue duration sessions.
No significant differences compared to saline were found for any of the parameters.
CNO5: CNO 5 mg/kg. CNO10: CNO 10 mg/kg injection.
Data are expressed as mean ± SD.

		dmPFC→DMS			vmPFC→VMS			eYFP		
Parameter	Sess. type	Saline	CNO_5	CNO_{10}	Saline	CNO_5	CNO_{10}	Saline	CNO_5	CNO_{10}
Number of started trials	Var delay	400.20 ±58.69	399.50 ±79.56	416.60 ±55.97	425.33 ±55.99	442.10 ±33.32	437.83 ±42.50	433.08 ±53.41	442.08 ±33.32	430.08 ±46.57
Magazine latency (s)	Var delay	2.15 ±0.36	2.04 ±0.38	2.03 ±0.37	2.27 ±0.33	2.28 ±0.35	2.27 ±0.30	2.24 ±0.28	2.28 ±0.35	2.30 ±0.34
Perseve-rative responses on target (%)	Var delay	2.98 ±2.21	2.91 ±1.66	3.04 ±1.11	3.24 ±1.56	2.99 ±1.48	2.70 ±1.15	3.12 ±1.55	2.99 ±1.48	2.82 ±1.22
Number of started trials	Var cue	432.3 ±84.11	416 ±107.95	431.7 ±75.71	424.58 ±103.31	415.67 ±105.65	424.75 ±123.73	517.25 ±48.07	509.42 ±37.65	505.25 ±42.98
Magazine latency (s)	Var cue	2.17 ±0.35	2.14 ±0.34	2.21 ±0.42	2.07 ±0.34	2.04 ±0.39	2.03 ±0.33	2.46 ±0.29	2.43 ±0.31	2.54 ±0.33
Perseve-rative responses on target (%)	Var cue	1.41 ±0.75	2.22 ±1.40	2.16 ±1.38	2.73 ±2.47	2.27 ±1.13	2.57 ±1.81	3.09 ±1.50	2.89 ±1.27	2.99 ±0.81
