# Supplementary material for: Bi-directional regulation of cognitive control by distinct prefrontal cortical output neurons to thalamus and striatum
Source: Nat Commun. 2021 Mar 31;12:1994. doi: 10.1038/s41467-021-22260-7 (PMC8012364; doi:10.1038/s41467-021-22260-7)
Supplement: Supplementary file 3 — Reporting Summary [file 41467_2021_22260_MOESM3_ESM.pdf]

nature research

Corresponding author(s): Sybren Friso de Kloet

Last updated by author(s): Dec 23, 2020

Reporting Summary

Nature Research wishes to improve the reproducibility of the work that we publish. This form provides structure for consistency and transparency in reporting. For further information on Nature Research policies, see our Editorial Policies and the Editorial Policy Checklist.

Statistics

For all statistical analyses, confirm that the following items are present in the figure legend, table legend, main text, or Methods section.

n/a	Confirmed	
☐	☒	The exact sample size (n) for each experimental group/condition, given as a discrete number and unit of measurement
☐	☒	A statement on whether measurements were taken from distinct samples or whether the same sample was measured repeatedly
☐	☒	The statistical test(s) used AND whether they are one- or two-sided<br>*Only common tests should be described solely by name; describe more complex techniques in the Methods section.*
☒	☐	A description of all covariates tested
☐	☒	A description of any assumptions or corrections, such as tests of normality and adjustment for multiple comparisons
☐	☒	A full description of the statistical parameters including central tendency (e.g. means) or other basic estimates (e.g. regression coefficient) AND variation (e.g. standard deviation) or associated estimates of uncertainty (e.g. confidence intervals)
☐	☒	For null hypothesis testing, the test statistic (e.g. F, t, r) with confidence intervals, effect sizes, degrees of freedom and P value noted<br>*Give P values as exact values whenever suitable.*
☒	☐	For Bayesian analysis, information on the choice of priors and Markov chain Monte Carlo settings
☒	☐	For hierarchical and complex designs, identification of the appropriate level for tests and full reporting of outcomes
☐	☒	Estimates of effect sizes (e.g. Cohen's d, Pearson's r), indicating how they were calculated

Our web collection on statistics for biologists contains articles on many of the points above.

Software and code

Policy information about availability of computer code

Data collection	MED-PC IV (MedAssociates)<br>Synapse s3 (TDT)<br>pClamp 11 (Molecular Devices)<br>Eclipse Ti software (Nikon)<br>Neuromantic (V1.6.3)
Data analysis	Matlab 2019b (MathWorks)<br>Prism 6 (GraphPad)<br>ImageJ (NIH)<br>Code: https://zenodo.org/record/4568820

For manuscripts utilizing custom algorithms or software that are central to the research but not yet described in published literature, software must be made available to editors and reviewers. We strongly encourage code deposition in a community repository (e.g. GitHub). See the Nature Research guidelines for submitting code & software for further information.

Data

Policy information about availability of data

All manuscripts must include a data availability statement. This statement should provide the following information, where applicable:

- Accession codes, unique identifiers, or web links for publicly available datasets
- A list of figures that have associated raw data
- A description of any restrictions on data availability

Data availability statement in the manuscript: "All datasets are available upon reasonable request."

Field-specific reporting

Please select the one below that is the best fit for your research. If you are not sure, read the appropriate sections before making your selection.

- [x] Life sciences
- [ ] Behavioural & social sciences
- [ ] Ecological, evolutionary & environmental sciences

For a reference copy of the document with all sections, see nature.com/documents/nr-reporting-summary-flat.pdf

Life sciences study design

All studies must disclose on these points even when the disclosure is negative.

Sample size	Sample sizes were determined based on power analysis, with expected variance and effect sizes based on literature (Chemogenetics/behavior: e.g. Luchicchi et al., 2016, PMID 27630545; Koike et al., 2015, PMID 26224620 - Photometry: e.g. Kupferschmidt et al., 2017, PMID 29024667; Pisansky et al., 2019, PMID 31471038) and experience
Data exclusions	Animals with mistargeted viral expression (e.g. in case of fiber photometry, where there were no GCaMPm-expressing neurons under the fiber tip in the area where we would expect signal, as shown in Supplementary Figures or Kupferschmidt et al., 2017, PMID 29024667), without any virus expression, or with optic fiber implants not in the appropriate brain region were excluded in fiber photometry experiments. In the chemogenetics experiments, animals with unilateral or no DREADD expression were excluded. Prior perturbation studies in the 5-CSRTT have often chosen for bilateral manipulations (e.g Chudasama et al., 2003, PMID 14643464, or Koike et al., 2016, PMID 26224620), we chose to remain consistent with previous literature, especially in experiments where behavioral performance is the read-out. For neuroanatomical experiments, animals with mistargeted retrobead injections were excluded.
Replication	We validated the CombiCage so that behavioral performance would be stable and comparable to conventional 5-CSRTT (published in Bruinsma et al., 2019, PMID 30826849). Behavioral training and experiments (photometry, chemogenetics) were replicated by 3 different researchers with consistent outcomes. Slice electrophysiology experiments were performed by 2 different researchers with consistent outcomes. Virus injections for all experiments (photometry, chemogenetics, tracing, and ephys) were done by 4 different researchers yielded consistent outcomes in viral targeting and expression. Fiber implants were performed by two different researchers, targeting was consistent.
Randomization	In behavioral experiments, animals were randomly allocated to a 'projection target' group. CNO injection doses were varied randomly, as were variable delay and stimulus duration sessions. Within the sessions, delay and cue durations were varied pseudorandomly (so that the session would end up with a relatively similar number of trials in each condition).
Blinding	Blinding was not possible this study. Virus injections and implantations required highly targeted injections, precluding any blinding.

Reporting for specific materials, systems and methods

We require information from authors about some types of materials, experimental systems and methods used in many studies. Here, indicate whether each material, system or method listed is relevant to your study. If you are not sure if a list item applies to your research, read the appropriate section before selecting a response.

Materials & experimental systems

n/a	Involved in the study	
[ ]	[x]	Antibodies
[x]	[ ]	Eukaryotic cell lines
[x]	[ ]	Palaeontology and archaeology
[ ]	[x]	Animals and other organisms
[x]	[ ]	Human research participants
[x]	[ ]	Clinical data
[x]	[ ]	Dual use research of concern

Methods

n/a	Involved in the study	
[x]	[ ]	ChIP-seq
[x]	[ ]	Flow cytometry
[x]	[ ]	MRI-based neuroimaging

Antibodies

Antibodies used	We used the following antibodies: mouse anti-NeuN (Abcam, Cat#104224, concentration 1:1000) with Alexa Fluor 647 donkey anti-mouse (Cat#15980296, Thermo Fisher Scientific, 1:400), rabbit anti-RFP (Cat#600401379, Rockland, 1:1000) with Alexa Fluor 546 donkey anti-rabbit (Cat#10593125, Thermo Fisher Scientific, 1:400), mouse anti-GAD-67 (Cat#5406, Millipore, 1:1000) with Alexa Fluor 647 donkey anti-mouse (Cat#15980296, Thermo Fisher Scientific, 1:400), and rabbit anti-GFP (Abcam, 1:1000) with Alexa Fluor 488 donkey anti-rabbit (Cat#10424752, Thermo Fisher Scientific, 1:400)
Validation	mouse anti-NeuN: 294 references (ex. PMID 31552908)<br>rabbit anti-RFP: >100 references (ex. PMID 32332079)<br>mouse anti-GAD-67: 193 references (ex. PMID 24723034)<br>rabbit anti-GFP: 894 references (ex. PMID 31859030)<br>Additionally, we included specific controls for each staining session (staining without either primary or secondary Ab)

Animals and other organisms

Policy information about studies involving animals; ARRIVE guidelines recommended for reporting animal research

Laboratory animals	Long Evans Rats, male, 6-32 weeks old
Wild animals	No wild animals were used in this study.
Field-collected samples	No field-collected samples were used in this study.
Ethics oversight	All experimental procedures were in accordance with European and Dutch law and approved by the animal ethical care committees of the VU University and VU University Medical Center, Amsterdam.

Note that full information on the approval of the study protocol must also be provided in the manuscript.
